# The Synthesis and Antitumor Activity of the Sodium Salt and Copper (II) Complex of N-[(Trimethylamineboryl)-Carbonyl]-L-Phenylalanine Methyl Ester

**DOI:** 10.1155/MBD.1998.1

**Published:** 1998

**Authors:** M. C. Miller, A. Sood, B. F. Spielvogel, I. H. Hall

**Affiliations:** 1 Division of Medicinal Chemistry & Natural Products School of Pharmacy University of North Carolina Chapel Hill NC 27599-7360 USA; 2 Boron Biologicals, Inc. 620 Hutton St. Raleigh NC 27606 USA

## Abstract

Sodium N-[(trimethylamineboryl)-carbonyl]-L-phenylalanine **2** and {N-[(trimethylamineboryl)-carbonyl]-L-phenylalanyl- carbxylato}-bis-{N-[(trimethylaminebryl)-carbonyl]-L-phenylalanine} dicopper (II) **3** were successfully synthesized. The agents blocked L_1210_ leukemic cell DNA and RNA syntheses by inhibiting multiple enzyme activities for nucleic acid synthesis, e.g. PRPP amido transferase, IMP dehydrogenase, DNA polymerase α, thymidine kinase, and TMP kinase. The copper (II) complex **3** demonstrated improved ability to inhibit L_1210_ partially purified DNA topoisomerase II compared to the parent compound while the sodium salt was inactive at 100 μM.


### THE SYNTHESIS AND ANTITUMOR ACTIVITY OF THE SODIUM SALT AND COPPER (II) COMPLEX OF N-[(TRIMETHYLAMINEBORYL)-CARBONYL]-L-PHENYLALANINE METHYL ESTER

Miller, III, M. C.[1], Sood, A.[2], Spielvogel, B. F.[2], and Hall, I. H.*[1]

[1] Division of Medicinal Chemistry & Natural Products, School of Pharmacy, University of North Carolina, Chapel Hill, NC 27599-7360, USA
[2] Boron Biologicals, Inc., 620 Hutton St., Raleigh, NC 27606, USA

### ABSTRACT

Sodium N-[(trimethylamineboryl)-carbonyl]-L-phenylalanine **2** and {N-[(trimethylamineboryl)-carbonyl]-L-phenylalanyl-carboxylato}-bis-{N-[(trimethylamineboryl)-carbonyl]-L-phenylalanine} dicopper (II) **3** were successfully synthesized. The agents blocked $L_{1210}$ leukemic cell DNA and RNA syntheses by inhibiting multiple enzyme activities for nucleic acid synthesis, e.g. PRPP amido transferase, IMP dehydrogenase, DNA polymerase α, thymidine kinase, and TMP kinase. The copper (II) complex **3** demonstrated improved ability to inhibit $L_{1210}$ partially purified DNA topoisomerase II compared to the parent compound while the sodium salt was inactive at 100 μM.

### INTRODUCTION

N-[(Trimethylamineboryl)-carbonyl]-L-phenylalanine methyl ester **1** has been shown to be an effective antineoplastic agent [0]. Mode of action studies in $L_{1210}$ cells demonstrated that DNA and RNA syntheses were inhibited. Enzyme activities including PRPP amido transferase, IMP dehydrogenase, and DNA polymerase α were significantly inhibited by the phenylalanine derivative [1]. Preliminary studies with trimethylamine carboxyborane have shown that whereas the amine borane derivatives are potent antineoplastic agents [2], metal complexation of these agents with copper, cobalt, chromium, iron and zinc complexes, produced more potent derivatives [3,4]. Thus the following study involves comparison of the modes of action of the sodium salt and the copper (II) complex of N-[(trimethylamineboryl)-carbonyl]-L-phenylalanine in $L_{1210}$ cells.

### MATERIALS AND METHODS

### Chemistry

All chemicals were used as received from the manufacturer. Solvents were distilled prior to use. Trimethylamine carboxyborane was provided by Boron Biologicals, Inc. (Raleigh, NC). N-[(Trimethylamineboryl)-carbonyl]-L-phenylalanine methyl ester **1** was synthesized according to the method of Sood *et al.*[1]. All other chemicals used in syntheses were purchased from Aldrich Chemical Company (Milwaukee, WI). A Perkin Elmer 1320 Infrared Spectrophotometer was used for infrared (IR) analyses. IR spectra were obtained as KBr disks or as nujol mulls using sodium chloride plates. A Varian 300 MHz NMR spectrometer was used to generate $^1$H-NMR spectra. Chemical shifts are relative to the external standard tetramethylsilane (δ = 0). A Thomas-Hoover capillary melting point apparatus was used to determine melting points, which were uncorrected. Elemental analyses were performed by M-H-W Laboratories (Phoenix, AZ). Silica gel 60F 254 plates (silica gel on aluminum, Aldrich Chemical Company) were used for thin layer chromatography.

**Synthesis of Sodium N-[(trimethylamineboryl)-carbonyl]-L-phenylalanine (2):**

N-[(Trimethylamineboryl)-carbonyl]-L-phenylalanine methyl ester **1** (1 g, 3.59 mmol) was suspended in 50 ml distilled water to which 3.6 ml of an 0.986 M aqueous NaOH solution (0.14 g, 3.59 mmol) was slowly added while stirring. After 15 minutes, the reaction appeared to be complete by TLC analysis although small amounts of an insoluble white solid are present. The white solid was removed by vacuum filtration and solvent was removed under reduced pressure leaving a white hygroscopic solid. The white hygroscopic solid was further purified by silica gel column chromatography using one void volume of ethyl acetate, followed by ethyl acetate / acetonitrile (1:1) until completed. Combined pure fractions yielded 486.8 mg (47%) of a white hygroscopic solid; $R_f$=0.12 in 1:1 ethyl acetate/acetonitrile. $^1$H-NMR (DMSO-$d_6$): δ 7.16 (m,5H,$C_6H_5$); δ 6.57 (d, J= 9 Hz, 1H, NH); δ 4.35 (dd, J = 18, 9 Hz, 1H,CH); δ 3.10 (dd J = 13,4 Hz, 1H,$CH_2$); δ 2.88 (dd, J = 13,4 Hz, 1H, $CH_3$); δ 2.60 (s, 9H,$NMe_3$); δ 1.72 (m,2H,$BH_2$); IR $(cm^{-1})_{Nujol}$ : 3450 $\nu_{NH}$, 2380 $\nu_{BH}$, 1575 $\nu_{CONH}$; m.p. 95-97°C (dec.). Calcd.: C, 54.57%; H, 7.05%; N, 9.79%. Found: C, 54.33%; H, 6.95%; N, 9.57%.

**Synthesis of Tetrakis - μ - {N - [(trimethylamineboryl) - carbonyl] - L -phenylalanyl-carboxylato}- bis -{N -[(trimethylamineboryl)-carbonyl] -L-phenylalanine} dicopper (II) (3):**

A column (300 x 10 mm) was packed with IR-120 cation exchange resin to a bed volume of 10 ml by the method of McCubbins [5]. The column packing was washed with water (D.I., 4 x 25 ml) then charged with

HCl (1M, 4 x 25 ml). The column was washed with water (D.I., 25 ml) to remove the excess acid from the packing. A solution of copper (II) chloride (1M, 4 x 25 ml) was allowed to pass through the column. This was followed by washing the excess copper solution off of the column with water (D.I.) until sample eluent gave a negative chloride test result using 1% $AgNO_3$. The solvent in the column was changed from water to MeOH by using a gradient, increasing the MeOH content by 10% each 25 ml until 25 ml 100% methanol was eluted. Sodium N-[(trimethylamineboryl)-carbonyl]-L-phenylalanine (1) (100 mg) was dissolved in dry MeOH (1 ml) and eluted through the column. After elution of approximately 10 ml of MeOH, the clear mobile phase turned blue. The solvent was removed by rotary evaporation, resulting in 84 mg (84% yield) of a blue solid. $^1H$-NMR ($CD_3OD$) δ 7.26 (m,30H, 6 $C_6H_5$); δ 4.87 (m,6H, 6 NH); δ 3.36 (m, 6H, 6 CH); δ 2.72 (m, 12H, 6 $CH_2$); δ 2.60 (m, 54H, 6 $NMe_3$); δ 2.10 (m, 12H, $BH_2$); IR ($cm^{-1}$)$_{Nujol}$: 3440 $\nu_{NH}$, 2380 $\nu_{BH}$, 1580 $\nu_{CONH}$; m.p. 109-111°C (dec.) Calcd.: C, 54.86%; H, 7.20%; N, 9.84%. Found: C, 54.62%; H, 6.96%; N, 9.61%.

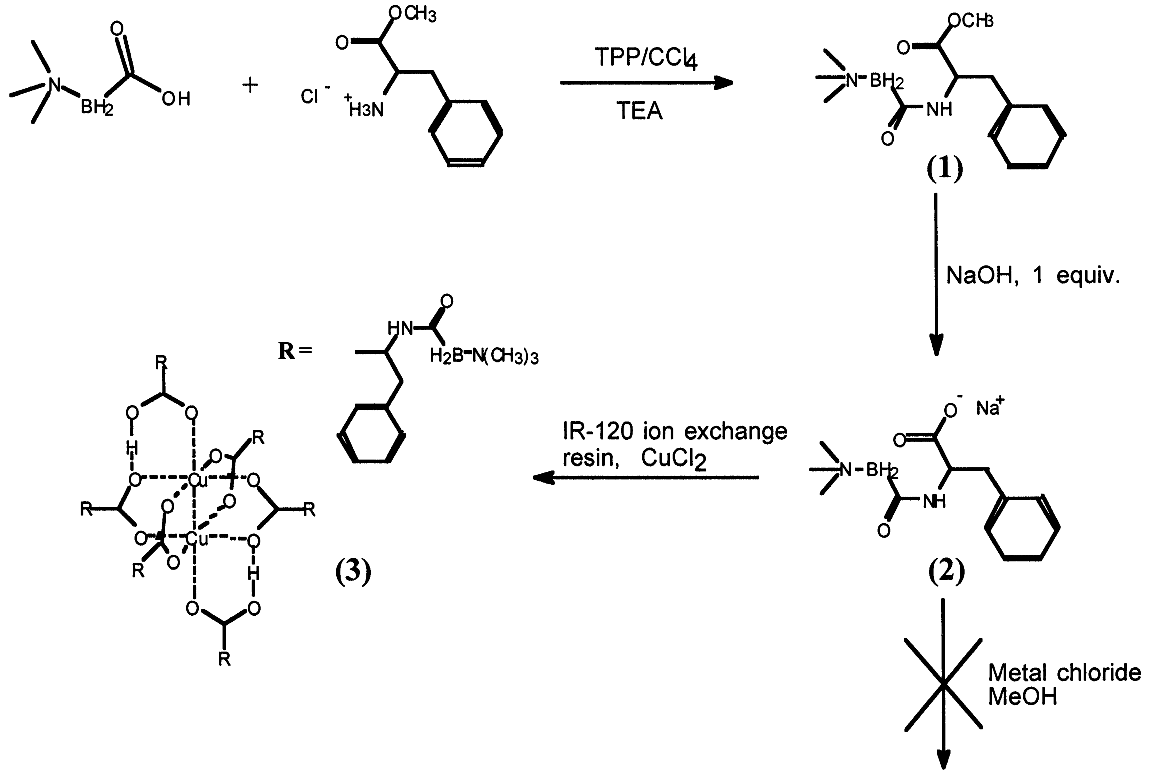

**Scheme 1:** The Synthesis of Derivatives of the Boronated Dipeptide N-[(Trimethylamineboryl)-carbonyl]-L-phenylalanine Methyl Ester.

**Cytotoxicity**

Compounds **1 - 3** were tested for cytotoxic activity by homogenizing drugs in a 1 mg/ml solution in 0.05% Tween 80/$H_2O$. These solutions were sterilized by passing them through an acrodisc (45 μm). The following cell lines were maintained by literature techniques [6]: murine $L_{1210}$ lymphoid leukemia, rat UMR 106 osteosarcoma, human $Tmolt_3$ acute lymphoblastic T cell leukemia, HeLa-$S^3$ suspended cervical carcinoma, HeLa solid cervical carcinoma, KB epidermoid nasopharynx, A431 epidermoid carcinoma, colorectal adenocarcinoma SW480, HCT-8 ileocecal adenocarcinoma, lung bronchogenic MB-9812, A549 lung carcinoma, and glioma HS683. Geran *et al.*'s protocol [7] was used to assess the suspended cell cytotoxicity of the compounds and standards in each cell line. Cell numbers were determined by the trypan blue exclusion technique. Solid tumor cytotoxicity was determined by Leibovitz *et al.'s* method [7] utilizing crystal violet/MeOH and read at 562 nm (Molecular Devices). Values for cytotoxicity were expressed as $ED_{50}$ = μg/ml, i.e. the concentration of the compound inhibiting 50% of cell growth. A value of less than 4 μg/ml was required for significant activity of growth inhibition.

**Incorporation Studies**

Incorporation of labeled precursors into $^3H$-DNA, $^3H$ -RNA and $^3H$ -protein for $10^6$ $L_{1210}$ cells was obtained [8] at concentration of the agents at 25, 50 and 100 μM during 60 min incubations. The incorporation of $^{14}C$ -glycine (53.0 mCi/mmol) into purines was obtained by the method of Cadman *et al.*[9]. Incorporation of $^{14}C$-formate (53.0 mCi/mmol) into pyrimidines was determined by the method of Christopherson *et al.*[10].

**Enzyme Assays**

Inhibition of various enzyme activities was performed by first preparing the appropriate $L_{1210}$ cell homogenates or subcellular fractions, then adding the drug to be tested during the enzyme assay. For the concentration response studies, inhibition of enzyme activity was determined at 25, 50 and 100 μM of compounds **2** and **3** after 60 min incubations. DNA polymerase α activity was determined in cytoplasmic extracts isolated by Eichler *et al.'s* method [11]. The polymerase assay DNA polymerase α was described by Sawada *et al.*[12] with $^3$H-TTP. Messenger-, ribosomal- and transfer-RNA polymerase enzymes were isolated with different concentrations of ammonium sulfate; individual RNA polymerase activities were determined using $^3$H -UTP [13,14]. Ribonucleoside reductase activity was measured using $^{14}$C -CDP with dithioerythritol [15]. The deoxyribonucleotides $^{14}$C -dCDP were separated from the ribonucleotides by TLC on PEI plates. Thymidine, TMP and TDP kinase activities were determined using $^3$H -thymidine (58.3 mCi/mmol) in the medium of Maley and Ochoa [16]. Carbamyl phosphate synthetase activity was determined with the method of Kalman *et al.*[17]; citrulline was determined colorimetrically [18]. Aspartate transcarbamylase activity was measured using the incubation medium of Kalman *et al.*[18]; carbamyl aspartate was determined colorimetrically by the method of Koritz *et al.*[19]. Thymidylate synthetase activity was analyzed by Kampf *et al.'s* method [20]. The $^3H_2O$ measured was proportional to the amount of TMP formed from $^3$H-dUMP. Dihydrofolate reductase activity was determined by the spectrophotometric method of Ho *et al.*[21]. PRPP amidotransferase activity was determined by Spassova *et al.'s* method [22]; IMP dehydrogenase activity was analyzed with 8-$^{14}$C-IMP (54 mCi/mmol) (Amersham, Arlington Heights, IL) after separating XMP on PEI plates (Fisher Scientific) by TLC [23]. Protein content was determined for the enzymatic assays by the Lowry technique [24].

Table 2: Effects of Sodium N-[(Trimethylamineboryl)-carbonyl]-L-phenylalanine (2) on $L_{1210}$ Leukemia Cell Metabolism *In Vitro* Over 60 Min. Percent of Control (mean ± s.d.) (N=4)

| Assay | Control | 25 μM | 50 μM | 100μM |
|---|---|---|---|---|
| DNA synthesis | 100±5[a] | 94±6 | 94±5 | 52±5* |
| RNA synthesis | 100±6[b] | 82±5 | 77±6* | 75±4* |
| Protein synthesis | 100±5[c] | 102±6 | 99±5 | 91±5 |
| DNA polymerase α | 100±6[d] | 69±6* | 58±5* | 49±4* |
| rRNA polymerase | 100±4[e] | 104±6 | 94±2 | 89±4 |
| mRNA polymerase | 100±7[f] | 99±5 | 91±3 | 68±4* |
| tRNA polymerase | 100±7[g] | 102±6 | 102±4 | 77±5* |
| Ribonucleoside diphosphate reductase | 100±5[h] | 111±5 | 99±5 | 96±3 |
| Dihydrofolatereductase | 100±5[i] | 127±9 | 112±5 | 88±5 |
| *De novo* purine synthesis | 100±5[j] | 42±5* | 29±5* | 28±3* |
| PRPP amido transferase | 100±6[k] | 84±5 | 58±4* | 51±3* |
| IMP dehydrogenase | 100±5[l] | 51±5* | 40±4* | 36±4* |
| Carbamyl phosphate synthetase | 100±7[m] | 105±7 | 98±5 | 95±6 |
| Aspartatetranscarbamylase | 100±6[n] | 106±5 | 94±6 | 92±5 |
| Thymidylate synthase | 100±5[o] | 99±6 | 98±5 | 77±6* |
| Thymidine kinase | 100±6[p] | 99±6 | 70±5* | 60±5* |
| TMP kinase | 100±7[q] | 91±5 | 50±4* | 33±3* |
| TDP kinase | 100±6[r] | 90±5 | 99±6 | 90±6 |
| d (ATP) pools | 100±5[s] | -- | -- | 109±5 |
| d (GTP) pools | 100±6[t] | -- | -- | 122±6* |
| d (CTP) pools | 100±5[u] | -- | -- | 104±8 |
| d (TTP) pools | 100±4[v] | -- | -- | 114±5 |

* $p \leq 0.001$

[a] 26152 dpm

[b] 4851 dpm

[c] 7461 dpm

[d] 47804 dpm

[e] 4239 dpm

[f] 1502 dpm

[g] 6400 dpm

[h] 2744 dpm

[i] 0.868 OD units

[j] 92551 dpm

[k] 0.121 OD units

[l] 76058 dpm

[m] 0.392 mol citrulline

[n] 1.064 mol N-carbamyl aspartate

[o] 18463 dpm

[p] 1317 dpm

[q] 1179 dpm

[r] 1891 dpm

[s] 6.17 pmol

[t] 5.27 pmol

[u] 6.87 pmol

[v] 6.94 pmol

**DNA Studies**

After deoxyribonucleoside triphosphates were extracted, [25] levels were determined by the method of Hunting and Henderson [26] with calf thymus DNA, *E. coli* DNA polymerase I, non-limiting amounts of the three deoxyribonucleoside triphosphates not being assayed, and either 0.4 mCi of ($^3$H-methyl)-dTTP or (5-$^3$H)-dCTP. The effects of compounds 2 and 3 on DNA strand scission was determined by the methods of Suzuki *et al.*[27], Pera *et al.*[28] and Woynarowski *et al.*[29]. $L_{1210}$ lymphoid leukemia cells were incubated with 10 µCi [methyl-$^3$H]-thymidine (84.0 Ci/mmol) for 24 hr at 37°C. $L_{1210}$ cells ($10^7$) were harvested and then centrifuged at 600 g X 10 min in PBS. They were later washed and suspended in 1 ml of PBS. Lysis buffer (0.5 ml; 0.5 M NaOH, 0.02 M EDTA, 0.01% Triton X-100 and 2.5% sucrose) was layered onto a 5-20% alkaline-sucrose gradient (5 ml; 0.3 M NaOH, 0.7 KCl and 0.01 M EDTA); this was followed by 0.2 ml of the cell preparation. After the gradient was incubated for 2.5 hr at room temperature, it was centrifuged at 12,000 RPM at 20°C for 60 min (Beckman rotor SW60). Fractions (0.2 ml) were collected from the bottom of the gradient, neutralized with 0.2 ml of 0.3 N HCl, and measured for radioactivity.

**Table 3**: Effects of Tetrakis-µ-{N-[(trimethylamineboryl)-carbonyl]-L-phenylalanyl-carboxylato}-bis-{N-[(trimethylamineboryl)-carbonyl]-L-phenylalanine} Dicopper (II) (3) on $L_{1210}$ Leukemia Cell Metabolism *In Vitro* Over 60 Min. Percent of Control (mean ± s.d.)
(N=4)

| Assay | Control | 25 µM | 50 µM | 100µM |
|---|---|---|---|---|
| DNA synthesis | 100±5[a] | 85±6 | 80±5* | 62±4* |
| RNA synthesis | 100±6[b] | 64±5* | 59±5* | 31±3* |
| Protein synthesis | 100±5[c] | 112±7 | 116±8 | 112±5 |
| DNA polymerase α | 100±6[d] | 73±5* | 65±6* | 46±5* |
| rRNA polymerase | 100±4[e] | 127±6* | 120±5 | 113±5 |
| mRNA polymerase | 100±7[f] | 82±5 | 79±6* | 70±4* |
| tRNA polymerase | 100±7[g] | 99±5 | 89±7 | 85±5 |
| Ribonucleoside diphosphate reductase | 100±5[h] | 102±5 | 99±6 | 90±6 |
| Dihydrofolatereductase | 100±5[i] | 133±6 | 80±6 | 59±5* |
| *De novo* purine synthesis | 100±5[j] | 106±6 | 67±5* | 38±4* |
| PRPP amido transferase | 100±6[k] | 67±7* | 44±4* | 31±4* |
| IMP dehydrogenase | 100±5[l] | 58±5* | 49±4* | 36±4* |
| Carbamyl phosphate synthetase | 100±7[m] | 110±6 | 106±5 | 104±6 |
| Aspartatetranscarbamylase | 100±6[n] | 97±6 | 97±5 | 95±6 |
| Thymidylate synthase | 100±5[o] | 104±5 | 92±5 | 78±5* |
| Thymidine kinase | 100±6[p] | 49±6* | 48±5* | 42±5* |
| TMP kinase | 100±7[q] | 42±6* | 31±5* | 28±4* |
| TDP kinase | 100±6[r] | 101±6 | 86±5 | 83±5 |
| d (ATP) pools | 100±5[s] | -- | -- | 104±4 |
| d (GTP) pools | 100±6[t] | -- | -- | 123±5* |
| d (CTP) pools | 100±5[u] | -- | -- | 104±5 |
| d (TTP) pools | 100±4[v] | -- | -- | 104±3 |

* p ≤ 0.001

[a] 26152 dpm

[b] 4851 dpm

[c] 7461 dpm

[d] 47804 dpm

[e] 4239 dpm

[f] 1502 dpm

[g] 6400 dpm

[h] 2744 dpm

[i] 0.868 OD units

[j] 92551 dpm

[k] 0.121 OD units

[l] 76058 dpm

[m] 0.392 mol citrulline

[n] 1.064 mol N-carbamyl aspartate

[o] 18463 dpm

[p] 1317 dpm

[q] 1179 dpm

[r] 1891 dpm

[s] 6.17 pmol

[t] 5.27 pmol

[u] 6.87 pmol

[v] 6.94 pmol

---

Thermal calf thymus DNA denaturation studies, changes in ct-DNA U.V. absorption from 220-340 nm, and ct-DNA viscosity studies were conducted after incubation of compounds **2** and **3** at 100 µM at 37°C for 24 hr [30].

$L_{1210}$ DNA-topoisomerase II was isolated by the method of Miller, *et al.*[31]. The 170 kDa topoisomerase II is present in the final preparation using the following procedures. All steps were carried out at 0-4°C. $L_{1210}$ cells (2 x $10^8$) were collected by centrifugation (500 x g for 5 min.), washed twice with PBS and resuspended in buffer solution containing 0.25 M sucrose, 20 mM potassium phosphate (pH 7.5), 2 mM $MgCl_2$, 1 mM

spermidine, 0.1 mM EDTA, 0.1 mM phenylmethyl sulfonyl fluoride (PMSF), and 1 mM $NaS_2O_5$ at 4°C. Cell membranes were lysed using a Dounce homogenizer following the addition of Triton X-100 at a volume equivalent to 1/100th the total cell suspension's volume.

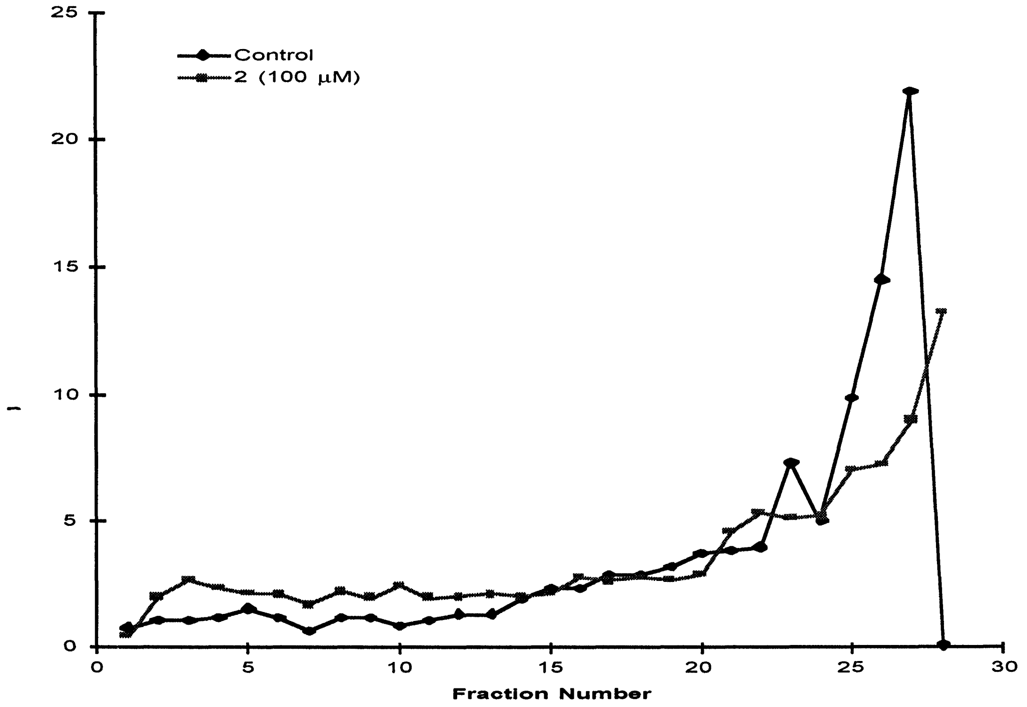

**Figure 1.** $L_{1210}$ DNA Strand Scission Induced by 24 Hour Incubation with Compound **2**.

Trypan blue staining of nuclei was used to determine complete cell lysis microscopically. An equal volume of buffer containing 1.75 M sucrose was added to the cell suspension and mixed by gently swirling. After mixing, the total volume was loaded on a sucrose cushion [1.4 M sucrose, 20 mM potassium phosphate (pH 7.5), 5 mM $MgCl_2$, 1 mM dithiothreitol (DTT), 0.1 mM EDTA, and 1 mM PMSF] and centrifuged at 18,000 rpm for 45 min at 4°C. The sucrose cushion was removed via vacuum aspiration and the remaining pellet was resuspended in buffer containing 20 mM potassium phosphate (pH 7.5), 2 mM $MgCl_2$, 1 mM dithiothreitol (DTT), 0.1 mM EDTA, and 1 mM PMSF, 1 mM β-mercaptoethanol, 10% glycerol, and 100 mM NaCl. The suspension was incubated at 4°C for 30 min. The process was repeated with buffers containing increasing concentrations of NaCl up to 400 mM. The supernatants containing enzyme activity were used for assays. The effects of compounds **2** and **3** on isolated DNA topoisomerase II activity was determined by the method of Miller *et al.*. Reactions containing 0.05 M Tris (pH 7.5), 0.1 M KCl, 0.01 M $MgCl_2$, 30 μg/ml bovine serum albumin, 0.5 mM EDTA, 1.0 mM DTT, 1.0 mM ATP, 0.1 μg knotted DNA (isolated by the method of Liu and Davis [32]), 1U $L_{1210}$ topoisomerase II and drugs were prepared. The samples were allowed to incubate at 37°C for 1 h and then stopped by the addition of stop buffer [50% w/v sucrose, 0.5% w/v sodium dodecylsulfate (SDS), and 0.25% w/v bromophenyl blue]. Each sample was run for 18 h using a 0.7% agarose gel, in electrophoresis buffer (pH 8.0) [90 mM Tris, 2 mM EDTA, 90 mM boric acid], on a Gibco BRL Horizon 11 X 14 electrophoresis apparatus at 23v. VP-16 (etoposide) was used as an internal standard inhibitor for DNA topoisomerase II assay. Photographs of gels were made by illumination of gels on a U.V. light table using Polaroid 667 film. Densitometric analysis was performed by the method of Hofmann, *et al.*[33] using a GS 300 Transmittance / Reflectance Scanning Densitometer and the GS 365 Densitometry Program (version 2) for personal computers (Hoefer Scientific Instruments, San Francisco, CA). Photographs of agarose gels were scanned by the densitometer, in reflectance mode, perpendicularly to the direction of DNA migration, aligned with the unknotted DNA bands. To standardize the quantification of unknotted DNA, known amounts of completely unknotted P4 DNA were subjected to electrophoresis and photographed.

**Table 1:** *In Vitro* Cytotoxicity of Derivatives of the Boronated Dipeptide N-[(Trimethylamineboryl)carbonyl]-L-phenylalanine Methyl Ester and Metal Complexes of Carboxyborane Adducts.

| $ED_{50}$ value (μg/ml)* | | | | | | | | | | | | |
|---|---|---|---|---|---|---|---|---|---|---|---|---|
| (N=6) | Murine | Rat | Human | | | | | | | | | |
| Cmpd | $L_{1210}$ Leukemia | UMR-106 Osteo-sarcoma | $Tmolt_3$ Leukemia | HeLa-$S^3$ Uterine Carcinoam | HeLa solid | KB Naso-pharnyns | SW480 Colon | HCT-8 Ileum | MB-9812 Lung | A549 Lung | A431 Skin | HS-683 Glioma |
| **1** | 3.34 | 5.96 | 1.31 | 4.01 | 3.33 | 1.99 | 8.19 | 8.13 | 5.78 | 10.84 | 4.20 | 6.88 |
| **2** | 2.68 | 4.00 | 4.22 | 2.37 | 1.82 | 8.02 | 4.29 | 5.13 | 1.33 | 2.97 | 5.83 | 14.2 |
| **3** | 4.31 | 3.24 | 6.38 | 1.91 | 5.92 | 4.46 | 3.22 | 4.46 | 3.84 | 10.04 | 2.39 | 8.72 |
| standard agents for comparison | | | | | | | | | | | | |
| 6-MP | 2.43 | 9.13 | 1.62 | 2.12 | 5.61 | 11.04 | 3.61 | 1.15 | 4.29 | 4.71 | 3.42 | 4.46 |
| Ara-C | 2.43 | 0.86 | 2.67 | 2.13 | 4.74 | 2.84 | 3.42 | 2.54 | 6.16 | 6.28 | 0.92 | 1.88 |
| Hydroxyurea | 2.67 | 2.87 | 4.47 | 1.96 | 8.12 | 5.27 | 7.33 | 1.77 | 7.18 | 8.89 | 3.21 | 2.27 |
| 5-FU | 1.41 | 3.52 | 2.14 | 2.47 | 4.11 | 1.25 | 3.09 | 1.12 | 5.64 | 3.58 | 0.61 | 1.28 |
| VP-16 | 1.83 | 3.57 | -- | 7.87 | 3.05 | 3.32 | 3.34 | 3.78 | 3.50 | 4.74 | 0.71 | 2.44 |

* Significant $ED_{50}$ values were less than 4 μg/ml.

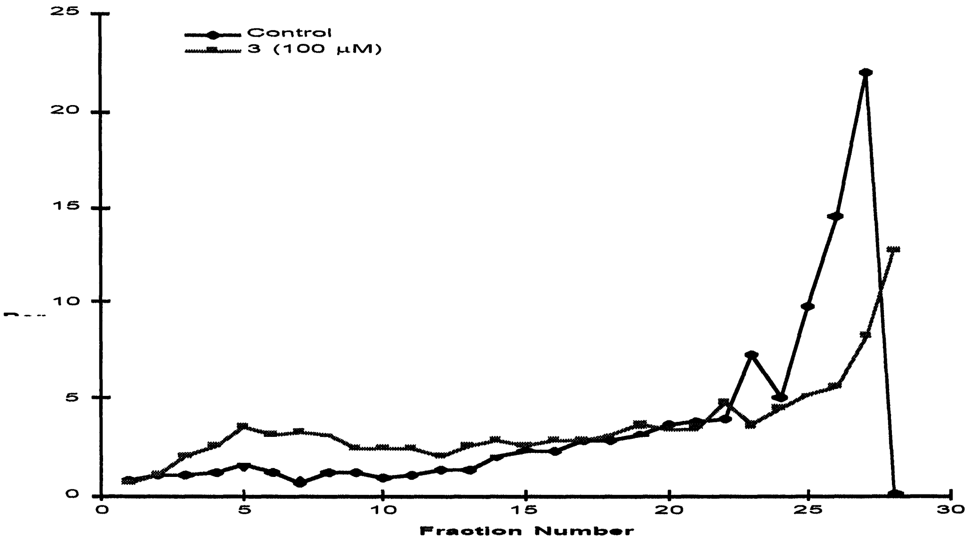

**Figure 2.** $L_{1210}$ DNA Strand Scission Induced by 24 Hour Incubation with Compound **3**.

The area under the curves corresponding to unknotted DNA bands were calculated using the densitometry software package. Data were plotted as percent of enzyme control. $IC_{50}$ values were calculated by non-linear regression analysis of plotted data using Prism®, version 2 (GraphPad Software Inc., San Diego, CA).

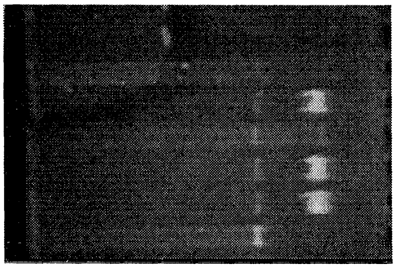

**Figure 3:** The Inhibition of $L_{1210}$ Topoisomerase II Activity *In Vitro* by Dipeptides and Metal Complexes of Amine Carboxyborane Adducts.

**Statistical Analysis**
Data as percentage of control is displayed in tables and figures as the means ± standard deviations. N is the number of samples per group. The Student's "t"-test was used to determine the probable level of significance (p) between test samples and control samples.

### RESULTS AND DISCUSSION

**Chemistry**
The sodium salt **2** was quickly formed in the presence of sodium hydroxide. The production of side products was noted which contributed to the lower than expected yield. The copper (II) complex was successfully synthesized using IR-120 cation exchange resin charged with the desired cation. Ion exchange proceeded by elution of a solution of the sodium salt **2** through a column of ion exchange resin. The structures and purity of the compound were confirmed and elemental analysis, melting points, and both $^{1}H$-NMR and infrared spectroscopy are reported. Attempts to form complexes by mixing methanolic solutions of carboxyborane sodium salts with methanolic solutions of metal chloride salts were unsuccessful. The synthesis of sodium N-[(trimethylamine-boryl)}-L-phenylalanine using sodium hydroxide provided an adequate yield of 47.4%; however, the appearance of additional reaction products was apparent by TLC. It is possible that the reaction conditions in the presence of sodium hydroxide may be caustic. After the initial reaction, only a slight amount of starting material and a large proportion of product were visible by TLC. Column chromatography proved a greater problem in that two impurities with similar $R_f$ values with respect to the desired product were apparent in the related fractions. It was not possible to remove these impurities by silica gel column chromatography as more of these impurities were generated with subsequent elutions. The formation of impurities during silica gel chromatography was possibly due to the acidity of the stationary phase which may facilitate protonation and rearrangement of the carboxyborane to boric acid as described by Sood, *et al.* [36], or hydrolysis of the amide and/or ester linkages in the dipeptide.

Initial attempts to synthesize metal complexes of carboxyborane adducts followed the method of Norwood et al. [37,38] in which the methanolic solution of sodium salts were added to methanolic solutions of metal chlorides. When applied to the study, a mixture of impurities were present at each stage of Norwood's procedure and purification was not possible. However, the use of IR-120 cation exchange resin provided a more successful means to synthesis tetrakis-μ-{N-{(trimethylamineboryl)-carbonyl-L-phenylalanine-carboxylato}-bis-[N - (trimethylamine-boryl) - carbonyl] - L]phenylalanine} dicopper(II) **3** without the need for further purification. The synthesis of other metal complexes using ion exchange resin was not pursued due to the limited amount of starting materials remaining after multiple attempts at metal complexation using the Norwood method.

**Antitumor Activity**

Synthesized compounds demonstrated *in vitro* cytotoxicity primarily in suspended tumor cell lines (e.g., murine $L_{1210}$ lymphoid leukemia, human $Tmolt_3$ T cell acute lymphoblastic leukemia, and HeLa-$S^3$ suspended human uterine cervical carcinoma) with variable activity in human solid tumor cell cultures. Murine $L_{1210}$ cytotoxicity assays demonstrated good activity for compound **2** ($ED_{50}$ = 2.68 μg/ml) which was more effective than the parent **1** ($ED_{50}$ = 3.34 μg/ml)[35]. Rat osteogenic sarcoma UMR-106 activity was noted for only compound **3** ($ED_{50}$ = 3.24 μg/ml). Against the growth of $Tmolt_3$ only the parent **1** was active ($ED_{50}$ = 1.31 μg/ml). Compounds **2** ($ED_{50}$ = 2.37 μg/ml) and **3** ($ED_{50}$ = 1.91 μg/ml) were active against human HeLa-$S^3$ suspended cervical carcinoma growth while the parent compound was inactive. In contrast compounds **1** ($ED_{50}$ = 3.38 μg/ml) and **2** ($ED_{50}$ = 1.82 μg/ml) were active against solid HeLa cervical carcinoma growth while compound **3** was inactive. The growth of KB nasopharynx was reduced by compound **1** ($ED_{50}$ = 1.99 μg/ml) alone. Only compound **3** ($ED_{50}$ = 3.22 μg/ml) effectively reduced SW480 colorectal adenocarcinoma cell growth. Against MB9812 bronchogenic lung growth, compounds **2** ($ED_{50}$ = 1.33 μg/ml) and **3** ($ED_{50}$ = 3.84 μg/ml) demonstrated activity while the parent was inactive. Compound **2** ($ED_{50}$ = 2.97 μg/ml) alone inhibited the growth of lung carcinoma A549, while only compound **3** ($ED_{50}$ = 2.39 μg/ml) was effective against A431 epidermoid carcinoma growth. None of the compounds tested were active against the growth of HCT-8 ileum or HS-683 glioma [Table 1].

The sodium salt and the copper (II) complex of N-[(trimethylamineboryl)-carbonyl]-L-phenylalanine were effective cytotoxic agents against the growth of a variety of rodent and human tumor cell lines. A wide variability was demonstrated with respect to which of these agents were the most active in a given screen. The copper (II) complex **3**, however, was not necessarily the most effective cytotoxic agent against each cell line. This indicated that metal complexation by the phenylalanine derivative does not necessarily improve cytotoxicity for certain cell lines.

Sodium N-[(trimethylamine- boryl)-carbonyl]-L-phenylalanine **2** and tetrakis-μ-{N-[(trimethylamineboryl) -carbonyl]-L-phenylalanyl-carboxylato}-bis-{N-[(trimethylamine-boryl)-carbonyl]-L-phenylalanine} dicopper (II) **3** were examined for their effects on $L_{1210}$ cell nucleic acid and protein metabolism at 25, 50, and 100 μM [Tables 2 and 3]. The synthesis of $L_{1210}$ DNA and RNA was significantly inhibited by compounds **2** and **3** in a concentration dependent manner, with maximal inhibition at 100 μM. DNA synthesis was reduced 48 and 38% by compounds **2** and **3**, respectively, at 100 μM. RNA synthesis was reduced 25 and 69% by compounds **2** and **3**, respectively, at 100 μM. Both compounds significantly inhibited DNA polymerase α activity at all concentrations employed, with greater than 50% inhibition at 100 μM. Compounds **2** and **3** were most effective in inhibiting purine *de novo* synthesis with a greater than 50% reduction at 25 μM for compound **2** and at 100 μM for compound **3**. Both PRPP amido transferase and IMP dehydrogenase activities were reduced greater than 50% at drug concentrations of 50 and 100 μM, respectively, by compound **2** and at 50 μM by compound **3** for both activities. These compounds were also able to affect thymidine kinase and TMP kinase activities causing significant reduction in both enzyme activities at 50 μM for compound **2** and greater than 50% reduction in both enzyme activities at 25 μM for compound **3**. These compounds did not affect $L_{1210}$ deoxynucleotide pool levels other than a significant increase in d(GTP) levels of 22% and 23% by compounds **2** and **3**, respectively, at 100 μM concentration.

Compounds **2** and **3** also demonstrated the ability to cause moderate $L_{1210}$ strand scission [Fig. 1 and 2]. This was indicated by the increase in low molecular weight (fragmented) DNA distributed throughout the alkaline sucrose gradients and a reduction of higher molecular weight double stranded DNA compared to the control. The ct-DNA U.V. absorption did not change after incubation for 24 hours with compounds **2** or **3**. This indicated no change in the helical structure of DNA and that the agents did not chemically interact with the DNA bases. There were no effects on U.V. absorption from 220 to 340 nm after 24 hour incubations with compounds **2** or **3**. This suggested that these agents do not alkylate DNA nucleotide bases. There was no change in the thermal denaturation $T_m$ values after 24 h incubation with the drugs, indicating no intercalation between DNA base pairs had occurred. The ct-DNA viscosity studies demonstrated less time was required to move through the reservoirs. Such an increase in flow rate suggested DNA viscosity was reduced by the agents. The ability of compound **2** and **3** to reduce DNA viscosity is consistent with their ability to cause moderate DNA strand scission.

Compounds **1 - 3** were evaluated for their mode of action as $L_{1210}$ DNA topoisomerase II inhibitors [Fig. 3]. Only the copper complex **3** demonstrated inhibitory activity at 100 μM concentration. Compound **3** demonstrated concentration dependent inhibitory effects on $L_{1210}$ topoisomerase II activity as evaluated by densitometry, affording an $IC_{50}$ value of 29 μM. In comparison the prototypical topoisomerase II inhibitor VP-16 which exhibited an $IC_{50}$ value of 22 μM.

In summary, mode of action studies on compound **2** primarily affected DNA synthesis at 100 μM while compound **3** had a greater effect on RNA synthesis. The *de novo* purine synthetic pathway appeared to be an important target for both compounds. Compound **2** preferentially reduced IMP dehydrogenase activity at 25 μM. In contrast, compound **3** markedly inhibited thymidine kinase and TMP kinase at 25 μM. At higher concentrations both compounds had their greatest effects on the purine pathway regulatory enzymes, PRPP amido transferase and IMP dehydrogenase, suggesting that inhibition of purine *de novo* synthesis is important in causing cytotoxicity. The magnitude of reduction afforded by compounds **2** and **3** on regulatory enzymes was sufficient to account for the observed reduction in DNA and RNA syntheses. In comparison, the parent compound **1**, also significantly reduced DNA and RNA syntheses. In contrast to compounds **2** and **3**, however, the parent compound had its greatest inhibitory effects on rRNA polymerase and ribonucleoside reductase activities [34]. The moderate effect of these compounds on double stranded DNA strand scission suggested that these compounds may cause DNA fragmentation but there was little evidence that the bases of DNA were the direct target of the agents. Copper (II) complexation to form compound **3** did improve the compound's ability to inhibit $L_{1210}$ DNA topoisomerase II activity compared to the parent compound which should improve the ability to inhibit DNA synthesis and cause cell death.

**References**

1. Sood, A., Sood, C.K., Spielvogel, B.F., Hall, I.H. *Eur. J. Med. Chem.* **25**, 301 (1990).
2. Hall, I.H., Hall, E.S., Miller, III, M.C., Sood, A., Spielvogel, B.F. *Amino Acids* **4**, 287 (1993).
3. Hall, I.H., Spielvogel, B.F., Sood, A. *Anti-Cancer Drugs* **1**, 133 (1990).
4. Hall, I.H., Spielvogel, B.F., McPhail, A.T. *J. Pharm. Sci.* **73**, 222 (1984).
5. Hall, I.H., Morse, K.W., Spielvogel, B.F., Sood, A. *Anti-Cancer Drugs* **2**, 389 (1991).
6. McCubbins, J.T. Ph.D. Dissertation, Duke University, (1990).
7. Leibovitz, A., Stinson, J.C., McCombs, III., W.B., McCoy, C.E., Mazur, K.C., Mabry, N.D. *Cancer Res.* **36**, 4562 (1976).
8. Geran, R.I., Greengerg, N.H., MacDonald, M.M., Schumacher, A.M., Abbott, B.J.*Cancer Chemo. Rep.* **3**, 7 (1972).
9. Liao, L.L., Kupchan, S.M., Horwitz, S.B. *Mol. Pharmacol.* **12**, 167 (1976).
10. Cadman, E., Heimer, R., Benz, C. *J. Biol. Chem.* **252**, 1695 (1981).
11. Christopherson, R.I., Yu, M.L., Jones, M.E. *Anal. Biochem.* **11**, 240 (1981).
12. Eichler, D.C., Fisher, P.A., Korn, D. *J. Biol. Chem.* **252**, 4011 (1977).
13. Sawada, H., Tatsumi, I., Sadada, M., Shirakawa, S., Nakamura, R., Wakisaka, G. *Cancer Res.* **34**, 3341 (1974).
14. Anderson, K.M., Mendelson, I.S., Guzik, G. *Biochem., Biophys. Acta* **383**, 56 (1975).
15. Hall, I.H., Carlson, G.L., Abernathy, G.S., Piantadosi, C. *J. Med. Chem.* **17**, 1253 (1974).
16. Moore, E.C., Hurlbert, R.B. *J. Biol. Chem.* **241**, 4802 (1966).
17. Maley, R., Ochoa, S. *J. Biol. Chem.* **233**, 1538 (1958).
18. Kalman, S.M., Duffield, P.H., Brzozuski *J. Biol. Chem.* **241**, 1871 (1966).
19. Archibald, R.M. *J. Biol. Chem.* **156**, 121 (1944).
20. Koritz, S.B., Cohen, P.P. *J. Biol. Chem.* **209**, 145 (1954).
21. Kampf, A., Barfknecht, R.L., Schaffer, P.J., Osaki, S., Mertes, M.P. *J. Med. Chem.* **19**, 903 (1976).
22. Ho, Y.K., Hakala, T., Zakrzewski, S.F. *Cancer Res.* **32**, 1023 (1972).
23. Spassova, M.K., Russev, G.C., Goovinsky, E.V. *Biochem. Pharmacol.* **25**, 923 (1976).
24. Becker, J.H., Lohr, G.W. *Klin. Wochenschr.* **57**, 1109 (1979).
25. Lowry, O.H., Rosebrough, J., Farr, A.L., Randall, R.J. *J. Biol. Chem.* **193**, 265 (1951).
26. Bagnara, A.S., Finch, L.R. *Anal. Biochem.* **45**, 24 (1971).
27. Hunting, D., Henderson, J.F. *Can. J. Biochem.* **59**, 723 (1982).
28. Suzuki, H., Nishimura, T., Muto, S.K., Tanaka, N. *J. Antibacteriol.* **32**, 875 (1978).
29. Pera, J.F., Sr., Rawlings, C.J., Shackleton, J., Roberts, J.J. *Biochem. Biophys. Acta* **655**, 152 (1981).
30. Woynarowski, J.W., Beerman, T.A., Konopa, J. *Biochem. Pharmacol.* **30**, 3005 (1981).
31. Zhao, Y., Hall, I.H., Oswald, C.B., Yokoi, T., Lee, K.H. *Chem. Pharm. Bull.* **35**, 2052 (1987).
32. Miller, K.G., Liu, L.F., Englund, P.T. *J. Biol. Chem.* **256**, 9334 (1981).
33. Liu, L.F., Davis, J.L. *Nucleic Acid Res.* **9**, 3979 (1981).
34. Hofmann, G.A., Mirabelli, C.K., Drake, F.H. *Anti-Cancer Drug Design* 5, 273 (1990).
35. Hall, I.H., Hall, E.S., Miller, III, M.C., Sood, A., Spielvogel, B.F. *Amino Acids* 4, 287 (1993).
36. Sood, A., Sood, C.K., Spielvogel, B.F., Hall, I.H., Wong O.T. *J. Pharm. Sci.* 81, 456 (1992).
37. Norwood V.M. III, Morse, K.W. *Inorg. Chem.* 25, 36990 (1986).
38. Norwood V.M. III, Morse, K.W. *Inorg. Chem.* 26, 284 (1987).

**Received: October 9, 1997 - Accepted: October 24, 1997 - Received in revised camera-ready format: December 8, 1997**

